# Effects of Sensitized Sorafenib with a Paeoniflorin and Geniposide Mixture on Liver Cancer via the NF-*κ*B-HIF-2*α*-SerpinB3 Pathway

**DOI:** 10.1155/2022/1911311

**Published:** 2022-10-15

**Authors:** Jun-Fei Li, Xiao-Rong Zheng, Hong-Yan Zhang, Chang-Ming Shen, Guo-xin Shen, Jian-Wei Jiang

**Affiliations:** The Cancer Hospital of the University of Chinese Academy of Sciences (Zhejiang Cancer Hospital), Institute of Basic Medicine and Cancer (IBMC), Chinese Academy of Sciences, Hangzhou, Zhejiang 310022, China

## Abstract

**Purpose:**

This study focused on determining the anticancer effect of paeoniflorin and geniposide mixture (PFGS) combined with sorafenib (Sor) in hepatocellular carcinoma (HCC) and, in particular, whether PFGS increases the antitumor effect of Sor by modulating the NF-*κ*B/HIF-2*α*/SerpinB3 pathway.

**Methods:**

The H22 hepatoma tumor-bearing mouse model was treated with PFGS, Sor, and a combination of the two drugs for 12 days. The effects of PFGS combined with Sor on tumor growth and apoptosis and the expression of NF-*κ*B, HIF-2*α*, and SerpinB3 in tumor tissue were assessed. In addition, Sor-resistant hepatoma cells were treated with PFGS, Sor, and the combination of the two drugs *in vitro*. The effects of PFGS combined with Sor on cell proliferation and invasion and the protein expression of NF-*κ*B p65, HIF-2*α*, and SerpinB3 were investigated.

**Results:**

PFGS combined with Sor treatment synergistically inhibited tumor growth in HCC tumor-bearing mice. Immunostaining showed that PFGS combined with Sor treatment significantly decreased the expression of Ki-67 and obviously induced apoptosis in the tumor compared with a single treatment. Similarly, PFGS combined with Sor treatment significantly downregulated the expression of NF-*κ*B, HIF-2*α*, and SerpinB3 in the tumor compared with a single treatment. Additionally, PFGS combined with Sor markedly inhibited cell proliferation and invasion and activation of the NF-*κ*B/HIF-2*α*/SerpinB3 pathway in Sor-resistant hepatoma cells compared with a single treatment.

**Conclusion:**

Our study demonstrated that PFGS synergistically increased the antiliver cancer effects of Sor by lowering activation of the NF-*κ*B/HIF-2*α*/SerpinB3 pathway. These findings provided a scientific foundation for clinical studies using PFGS and Sor to treat liver cancer.

## 1. Introduction

Hepatocellular carcinoma (HCC) has a high mortality rate and a proclivity for metastasis and recurrence. According to statistics, 900,000 patients were diagnosed with liver cancer, and there were 830,000 fatalities worldwide in 2020, making liver cancer one of the leading causes of cancer-related death [1]. Sorafenib (Sor) is a small molecule multikinase oral-targeted medication that has been shown to be effective in the first-line treatment of advanced primary liver cancer [2, 3]. However, Sor may induce drug resistance in HCC [4–6]. Relapse-free survival did not increase considerably in patients who received Sor for a long period in clinical studies, and the median survival time even deteriorated to various degrees [6–8], restricting the drug's use. Finding a safe and effective treatment to reduce drug resistance of liver cancer to Sor is therefore crucial for patients with this disease.

Sorafenib resistance in HCC, whether acquired or primary, necessitates abnormal expression of certain molecules or signaling pathways [7]. The nuclear factor kappa-B (NF-*κ*B) pathway is involved in the development and incidence of malignancies. The NF-*κ*B signaling cascade, when abnormally activated, can boost tumor cell proliferation and antiapoptosis, enhance epithelial-mesenchymal transition (EMT) and invasion, and develop treatment resistance [9]. Recent studies have shown that activation of NF-*κ*B was a key target in causing Sor desensitization in HCC [10]. Activated NF-*κ*B acts by inhibiting cytochrome P450 1A2 [11] or promoting the expression of CD47 [12] and hypoxia-induciblefactor-2*α* (HIF-2*α*) [13] and other pathways to induce the development of Sor resistance. The present findings provided a theoretical basis for increasing the efficacy of Sor in the treatment of liver cancer by blocking the NF-*κ*B signaling cascade.

Shaoyao Ruangan Mixture is a traditional Chinese medical preparation made by Zhejiang Cancer Hospital and has been used in the adjuvant treatment of hepatitis, HCC, and hepatic cirrhosis for more than 20 years. In a previous clinical application, the Shaoyao Ruangan Mixture conferred beneficial effects on chemically induced hepatic damage and effectively inhibited the progression of primary liver cancer [14, 15]. In addition, it was also found to inhibit the formation of tumor tissue in HepG2 tumor-bearing nude mice by suppressing NF-*κ*B and its downstream Bax/Bcl-2 and Caspase-3 [16]. The primary components of the mixture, according to drug analysis, are paeoniflorin (PF) and geniposide (GS), and in Shaoyao Ruangan Mixture at the concentration of 1 g/mL, there was approximately 0.235 mg/mL PF and 0.458 mg/mL·GS, with a content ratio of roughly 1 : 2 [17]. Recent investigations have also shown that PF and GS had considerable inhibitory effects on NF-*κ*B, and inhibiting NF-*κ*B expression could cause anti-inflammatory and antitumor effects [17–19]. We believe that the mixture can improve Sor sensitization in liver cancer due to the critical role of NF-*κ*B axis activation in Sor resistance. Therefore, to improve the antihepatoma effect of Sor, we used the paeoniflorin and geniposide mixture (PFGS), which was prepared based on the content ratio of the Shaoyao Ruangan Mixture. This was conducted to simplify the experiments and maintain the traditional Chinese medicine theory of increasing the effect with “assistance” (traditional Chinese medicine formulation) [20]^.^

We used H22 hepatoma tumor-bearing mice and a Sor-resistant human hepatoma cell line to investigate the antihepatoma effect of PFGS combined with Sor *in vivo* and *in vitro*, as well as the corresponding mechanism centered on the upstream and downstream target genes of the NF-*κ*B signaling pathway, laying the foundation for the clinical application of Shaoyao Ruangan Mixture and Sor.

## 2. Methods

### 2.1. Reagents

Paeoniflorin (PF), with a purity >98.04%, was purchased from MedChemExpress Inc. (Shanghai, China). Geniposide (GS), with a purity >99.52%, was purchased from MedChemExpress Inc. (Shanghai, China). The ratio of PF and GS was 1 : 2, which is close to the ratio of PF and GS in the Shaoyao Ruangan Mixture, which was used to prepare PFGS. Sorafenib, with a purity >99.08%, was purchased from MedChemExpress Inc. (Shanghai, China). Rabbit monoclonal antibodies against Ki-67 (ab15580), NF-*κ*B p65 (ab16502), HIF-2*α* (ab109616), and serine protease inhibitor B3 (SerpinB3, ab201081) were obtained from Abcam Inc. (Shanghai, China). A terminal deoxynucleotidyl transferase-mediated dUTP nick end-labeling (TUNEL) test kit was purchased from the Nanjing Jiancheng Biological Engineering Institute (Nanjing, China). Sodium dodecyl sulfate-polyacrylamide gel electrophoresis (SDS-PAGE), phosphate-buffered saline (PBS), PVDF membranes, BCA protein assay kit, penicillin/streptomycin (PS), dimethyl sulfoxide (DMSO), and 0.25% trypsin-0.53 mM EDTA were purchased from Solarbio Biotechnology Co., Ltd. (Beijing, China). Fetal bovine serum (FBS) was purchased from Hyclone Bioscience Co., Ltd. (Beijing, China). Dulbecco's Modified Eagle's Medium (DMEM) was purchased from Gibco Biotechnology Co., Ltd. (Beijing, China). Cell counting kit-8 (CKK-8) was obtained from Medchemexpress Inc. (Shanghai, China). BD Matrigel Basement Membrane Matrix (Matrigel) was obtained from Yes Service Biotech, Inc. (Shanghai, China).

### 2.2. Cell Line and Culture

The H22 cell line was purchased from Procell Life Science and Technology Co., Ltd. (Wuhan, China). Huh-7 and Huh-7Sor-resistant (SR) cell lines were purchased from iCell Bioscience Inc. (Shanghai, China). During resuscitation, the cells preserved in nitrogen were melted in a water bath at 37°C, a DMEM high sugar complete medium (89% DMEM high glucose medium + 10% fetal bovine serum + 1% penicillin/streptomycin) was added, and then it was centrifuged at 1000 rpm for 5 min to remove the supernatant. The precipitated cells were resuspended in a complete medium and inoculated into T25 culture flasks. The cells were maintained at a 37°C humidified incubator containing 5% CO_2_. The medium was replaced every 24 hours. When the cells were fused at about 80%, they were digested and passaged with pancreatin.

### 2.3. Animals and Treatment

A total of 24 male C57BL/6 mice, 5 weeks old and weighing 18–22 g, were obtained from SLAC Animal Inc. (Shanghai, China). The Animal Experiment Ethics Committee of Zhejiang Cancer Hospital approved all reported animal experiments (Registration No. SYKX 2017–0012, date of approval: 2017-10-10). All mice were housed in a controlled environment (12 h light/dark cycle, temperature of 22 ± 2°C and humidity of 45 ± 10%). After acclimatization, H22 cells (1 × 10^6^, viability ≥95%) were subcutaneously injected into the back or neck of mice to induce the H22 tumor-bearing mouse model. When the tumor grew to about 50 mm^3^, the mice were randomly divided into four groups (*n* = 6 in each group): control group (0.2 mL normal saline, stomach irrigation i. g., once a day), Sor group (30 mg/kg Sor, stomach i. g., once a day), PFGS group (25 mg/kg PF and 50 mg/kg·GS, stomach i. g., once a day), and PFGS-Sor group (30 mg/kg Sor, PF and 50 mg/kg·GS, stomach i. g., once a day). The dose of PF and GS in this experiment is closely equal to the content of PF and GS in the clinical dose Shaoyao Ruangan Mixture.

The tumor size was measured using calipers every 2 days, and the tumor volume was evaluated using the following formula: volume (mm^3^) = length (mm)× width (mm)× width (mm)/2. All intervention treatments lasted 12 days, and the mice were supplied with a standard rodent diet and water ad libitum during experimental periods. At the end of the 12 days of treatment, all surviving mice were euthanized by cervical dislocation, and the tumor tissue was removed immediately and weighed. The drug interaction coefficient (CDI) was computed as follows: *AB*/(*A *×*  B*). AB is the tumor weight of the combination/control group, A or B is the tumor weight of the single/control group, a CDI value less than, equal to, or greater than 1 represents synergy, additive, or antagonism, respectively, and CDI less than 0.7 indicates significant synergy.

### 2.4. Immunohistochemistry

The expression of Ki-67, NF-*κ*B, HIF-2*α*, and SerpinB3 in tumor tissue was detected by immunohistochemistry. After deparaffinization and rehydration, the samples were embedded and processed with trypsin for 10 min or heated for 25 min and subsequently incubated with a primary antibody overnight. After incubation with the primary antibody, a secondary antibody was added, and the sections were incubated for 30 min. Staining was developed using peroxidase 3,30-diaminobenzidine (DAB) substrate and counterstained with hematoxylin. The integrated optical density (IOD) and mean optical density (AOD) were quantified using Image-Pro Plus 6.0 software. AOD = IOD sum/area sum.

### 2.5. Tunel Analysis

Tissue paraffin sections were prepared, dewaxed in xylene, and hydrated in ethanol. The TUNEL reaction solution was then added according to the TUNEL kit instructions, followed by the DAB solution as the chromogenic substrate to each section. Finally, apoptosis was observed under a fluorescence microscope after rinsing with PBS. The ratio of apoptotic cells to total cells was quantified by Image-Pro Plus 6.0 software based on the IOD.

### 2.6. Cell Viability Assay

Cells in logarithmic growth were seeded into 96-well plates and cultured for up to 24 hours until adherence. Subsequently, PFGS or Sor or both were dissolved in DMSO (10 Mm), and the concentration of DMSO in the cell culture medium was <0.1%. Then, Huh-7 and SR cells were treated with different concentrations of Sor (0, 2.5, 5, 10, 20, 40 *μ*M); SR cells were treated with different concentrations of PFGS (0, 6, 12, 24, 48, 96 *μ*g/mL); and SR cells were treated with PFGS (6 or 12 *μ*g/mL) combined with different concentrations of Sor (0, 2.5, 5, 10, 20, 40 *μ*M) and incubated for 24 hours. Then, 10 *μ*L of CKK-8 solution was added to the cells, which were further incubated at 37°C for 4 hours. The optical density was then measured at 450 nm by a microplate reader (Varioskan Flash, Thermo) to calculate the IC_50_ and IC_10_ values. The percentage of cell viability was computed as follows: cell viability (%) = A450 (drug)/A450 (control)×100%. The drug interaction coefficient (CDI) was computed as follows: CDI = AB/(*A* × *B*). AB is the OD value of the combination/control group, A or B is the OD value of the single/control group, a CDI value less than, equal to, or greater than 1 represents synergy, additive, or antagonism, respectively, and CDI less than 0.7 indicates significant synergy.

### 2.7. Transwell Assay

The serum-free DMEM high glucose medium was diluted with Matrigel at the ratio of 3 : 1, and 30 *μ*L Matrigel diluent was placed in the Transwell chamber and incubated overnight at 4°C. Then, the Transwell chamber was placed into 24-well plates, and the cells in logarithmic growth were seeded into the upper chamber and cultured for up to 24 hours until adherence. Huh-7 and SR cells were treated with 5 *μ*M Sor or 12 *μ*g/mL PFGS or both and incubated for 24 hours. The cells in the bottom layer of the upper chamber were wiped off with a cotton swab, fixed with 4% paraformaldehyde for 10 min, washed three times with PBS, stained with 0.1% crystal violet for 30 min, and then photographed, and the number of cells invading the lower chamber were counted.

### 2.8. Western Blot Analysis

In brief, protein samples, which were extracted from Huh-7 or SR cells, were standardized using a BCA protein assay kit, loaded onto 8–12% SDS-PAGE, transferred to a PVDF membrane, and blocked with Tween-Tris-buffered saline (TBST) solution supplemented with 5% BSA. Subsequently, the membrane was incubated with a primary antibody at 4°C overnight. The next day, after washing with TBST, these membranes were incubated with a secondary antibody for 2 hours at room temperature, followed by enhanced chemiluminescence. Blotting was visualized using chemiluminescence (ChemiScope 3000 mini, Clinx Science Instruments Co., Ltd., Shanghai, China) following the manufacturer's instructions. *β*-Actin was selected as an internal control to compare protein levels. The intensity of the bands was determined based on Image J software.

### 2.9. Statistical Analysis

All values are expressed as the mean ± SEM. The Student's *t*-test was used for the comparison between groups and one-way ANOVA for the comparison of multiple groups. A value of *p* < 0.05 was considered to indicate a significant difference. GraphPad Prism 8.0 was used to analyze all statistical data.

## 3. Results

### 3.1. Antitumor Effect of PFGS Combined with Sor in H22 Hepatoma Tumor-Bearing Mice

**Figure 1:**
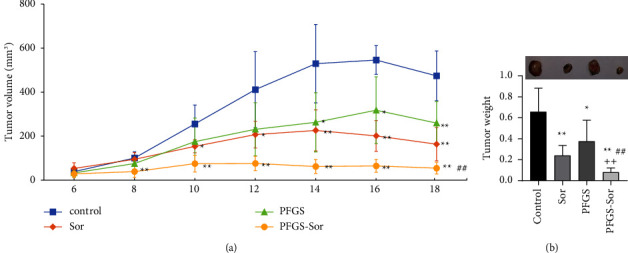
The antitumor effect of PFGS combined with Sor in H22 tumor-bearing mice. (a) PFGS combined with Sor showed significant inhibition of tumor volume (*n* = 6). (b) PFGS combined with Sor showed significant inhibition of tumor weight. Data are presented as the mean ± SD. ^*∗*^*p* < 0.05 compared with the control group; ^*∗∗*^*p* < 0.01 compared with the control group; ^##^*p* < 0.01 compared with the Sor group; ++ CDI<0.7 had a significant synergistic effect.

Following 12 days of treatment with PFGS combination with Sor, the tumor weight was significantly decreased in the Sor, PFGS, and PFGS combined with Sor groups compared with that in the control group. The tumor weight was also significantly lower in the PFGS combined with the Sor group compared with the Sor group, and the CDI value was 0.59 (Figure 1(b)). In addition, tumor growth in the control group was rapid, whereas tumor growth in the Sor or PFGS or PFGS combined with Sor treated groups was delayed. However, tumor volume was higher in the Sor and PFGS groups compared with that in the PFGS combined with the Sor group. Thus, compared with the control group, the Sor, PFGS, and PFGS combined with Sor groups showed significant inhibition of tumor volume over time (Figure 1(a)). These results show that PFGS combined with Sor had a significant synergistic effect on antiliver cancer.

### 3.2. Effect of PFGS Combined with Sor on Proliferation and Apoptosis in H22 Hepatoma Tumor-Bearing Mice *In Vivo*

**Figure 2:**
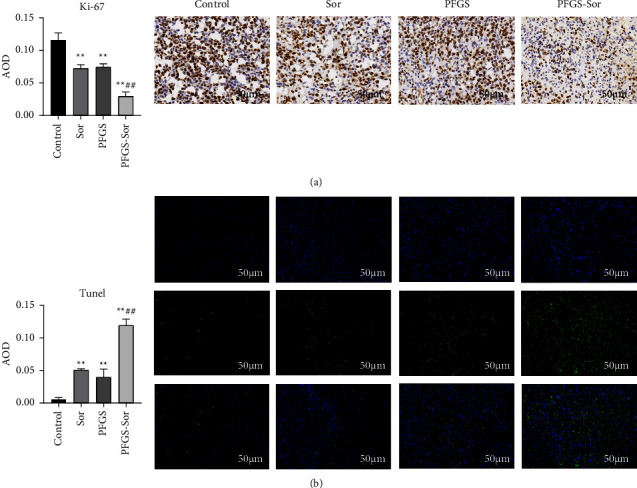
PFGS combined with Sor inhibited proliferation and triggered apoptosis in H22 tumor-bearing mice *in vivo*. (a) Immunostaining indicated that PFGS combined with Sor treatment markedly decreased the expression of Ki-67 (*n* = 3). (b) TUNEL staining showed that PFGS combined with Sor treatment increased the number of apoptotic cells in the tumor compared to the control group (*n* = 3). Data are presented as the mean ± SD. ^*∗*^*p* < 0.05 compared with the control group; ^*∗∗*^*p* < 0.01 compared with the control group; ^##^*p* < 0.01 compared with the Sor group.

We performed immunohistochemical staining to evaluate the expression of cell proliferative marker (Ki-67) in the tumor tissues of tumor-bearing mice. Brown staining indicates areas of positive expression, the shade of the color represents the expression level of the target protein, and the cell nuclei were stained blue by hematoxylin. Ki-67 was positively expressed in the control group, whereas in the Sor, PFGS, and PFGS combined with Sor treatment groups, the expression of Ki-67 markedly decreased in tumors. However, the expression of Ki-67 was obviously lower in the PFGS combined with Sor group compared with the Sor group (Figure 2(a)). Moreover, the number of TUNEL-positive cells was increased in the Sor, PFGS, and PFGS combined with the Sor groups compared with the control group, and the number of apoptotic cells was higher in the PFGS combined with the Sor group compared with the Sor group (Figure 2(b)). Thus, PFGS enhanced the effect of Sor in terms of proliferation inhibition and triggered apoptosis in H22 tumor-bearing mice *in vivo*.

### 3.3. Expression of NF-*κ*B, HIF-2*α*, and SerpinB3 in Tumor Tissues

**Figure 3:**
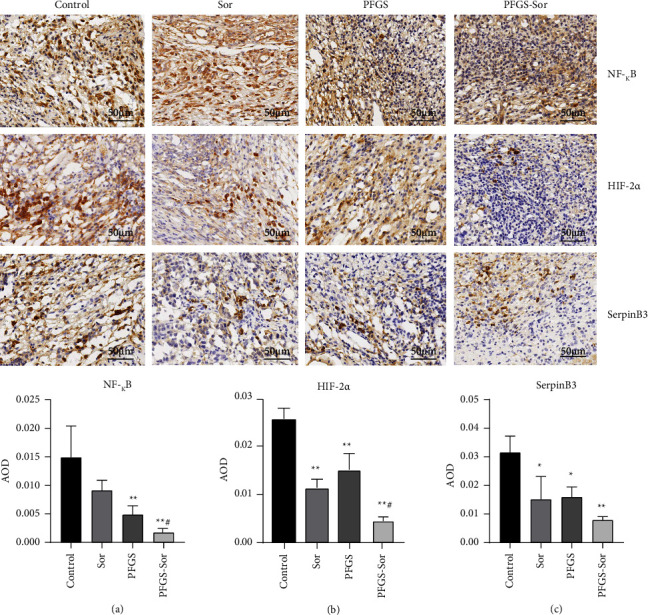
PFGS combined with Sor inhibited the expression of NF-*κ*B, HIF-2*α*, and SerpinB3 in H22 tumor-bearing mice *in vivo*. (a) Immunostaining indicated that PFGS combined with Sor downregulated the expression of NF-*κ*B, HIF-2*α*, and SerpinB3 in tumor tissue (*n* = 3). (b–d) Statistics chart. Data are presented as the mean ± SD. ^*∗*^*p* < 0.05 compared with the control group; ^*∗∗*^*p* < 0.01 compared with the control group; ^#^*p* < 0.05 compared with the Sor group.

We performed immunohistochemical staining to evaluate the expression of NF-*κ*B, HIF-2*α*, and SerpinB3 in the tissues of tumor-bearing mice. Brown staining indicates areas of positive expression, the shade of the color represents the expression level of the target protein, and the cell nuclei were stained blue by hematoxylin. We found that the expression of NF-*κ*B, HIF-2*α*, and SerpinB3 was decreased in the Sor, PFGS, and PFGS combined with Sor groups compared with the control group (Figures 3(a)–3(c)). Furthermore, the expression of NF-*κ*B and HIF-2*α* in the PFGS combined with the Sor group was significantly lower than that in the Sor group (Figures 3(a) and 3(b)), and the expression of SerpinB3 in the PFGS combined with the Sor group was lower than that in the Sor group, but there are no significant difference between them (Figure 3(c)). These findings suggested that Sor had a similar but more limited effect on significantly decreasing the expression of NF-*κ*B, HIF-2*α*, and SerpinB3.

### 3.4. Detection of Resistance in SR Cells to Sorafenib

**Figure 4:**
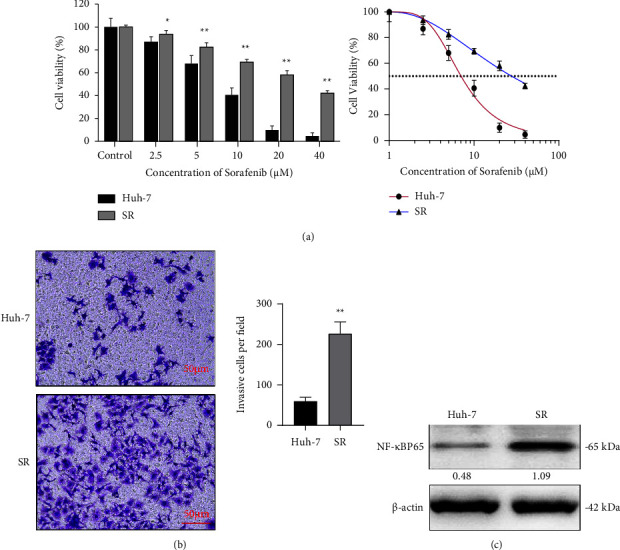
Drug resistance of SR cells. (a) Sor inhibited the viability of Huh-7 and SR cells in a dose-dependent manner. Cell viability was investigated using the CKK-8 assay (*n* = 6). (b) Invasion of Huh-7 and SR cells in the microporous membrane of the Transwell chamber (*n* = 3). (c) The protein expression of NF-*κ*B p65 in Huh-7 and SR cells (*n* = 3). Data are presented as the mean ± SD. ^*∗*^*p* < 0.05 compared with the Huh-7 group; ^*∗∗*^*p* < 0.01 compared with the Huh-7 group.

The effect of Sor on cell viability was evaluated in Huh-7 and SR cells at different concentrations (0, 2.5, 5, 10, 20, and 40 *μ*m) by the CKK-8 assay. The results showed that Sor had a dose-dependent effect on the viability of Huh-7 and SR cells. Notably, after incubation for 24 hours, the number of Huh-7 cells was significantly reduced compared to SR cells at each concentration of Sor. IC_50_ values were determined using GraphPad Prism (GraphPad Software Inc. San Diego, CA, USA.), and the IC_50_ values of Huh-7 and SR cells were 7.026 *μ*M and 27.733 *μ*M, respectively (Figure 4(a)). The effect of Sor on cell invasion ability was evaluated in Huh-7 and SR cells by Transwell assay, in which the chambers were covered was Matrigel. Following incubation for 24 hours, the invasion of SR cells treated with 5 *μ*m Sor, in the microporous membrane of the Transwell chamber was significantly lower compared to Huh-7 cells (Figure 4(b)). Moreover, the protein expression of NF-*κ*B p65 in Huh-7 cells was obviously lower than SR cells (Figure 4(c)). Thus, compared with Huh-7 cells, SR cells had greater resistance to Sor.

### 3.5. Antitumor Effect of PFGS Combined with Sor on SR Cells

**Figure 5:**
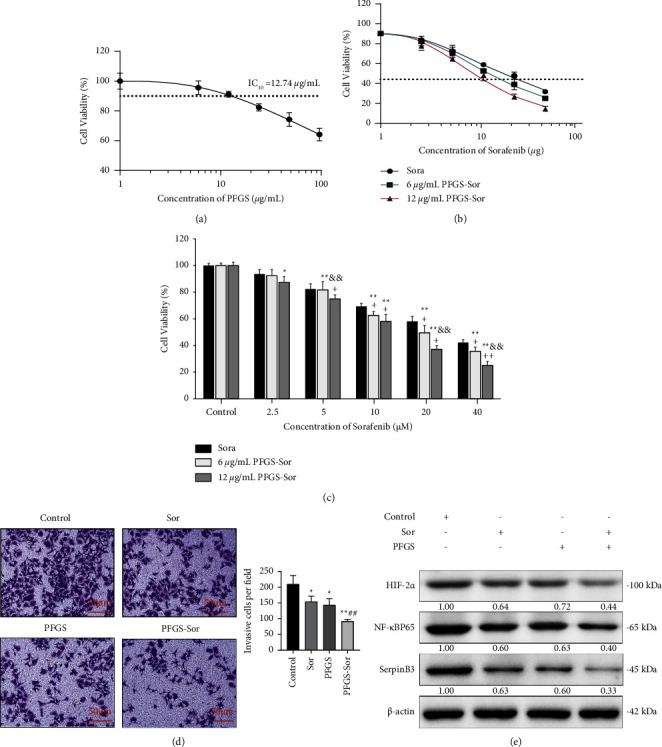
The antitumor effect of PFGS combined with Sor on SR cells. (a) PFGS inhibited the viability of SR cells in a dose-dependent manner. Cell viability was investigated using the CKK-8 assay (*n* = 6). (b, c) PFGS combined with Sor inhibited the viability of SR cells in a dose-dependent manner. Cell viability was investigated using the CKK-8 assay (*n* = 6). (d) Invasion of SR cells in the microporous membrane of the Transwell chamber (*n* = 3). (e) The protein expression of NF-*κ*B p65, HIF-2*α*, and SerpinB3 in SR cells (*n* = 3). Data are presented as the mean ± SD. ^*∗*^*p* < 0.05 compared with the control group; ^*∗∗*^*p* < 0.01 compared with the control group; ^&&^*p* < 0.01 compared with the 6 *μ*g/mL PFGS combined with the same concentration of Sor group; ^##^*p* < 0.01 compared with the control group; ^+^CDI<1 had a synergistic effect;^++^CDI<0.7 had a marked synergistic effect.

The effect of PFGS on cell viability was evaluated in SR cells at different concentrations (0, 6, 12, 24, 48, 96 *μ*g/mL) by the CKK-8 assay. The results showed that PFGS had a dose-dependent effect on the viability of SR cells, and the maximal nontoxic concentration of PFGS was 12.74 *μ*g/mL (IC_10_) (Figure 5(a)). As a result, we used 6 *μ*g/mL or 12 *μ*g/mL PFGS combined with different concentrations of Sor (0, 2.5, 5, 10, 20, and 40 *μ*m) to study the effect on cell viability. Following incubation for 24 h, the dose-dependent effect of Sor on the viability of SR cells with 6 *μ*g/mL or 12 *μ*g/mL PFGS was determined. The IC_50_ values of Sor, 6 *μ*g/mL PFGS combined with Sor, and 12 *μ*g/mL PFGS combined with Sor treatment in SR cells were 27.733 *μ*M, 18.493 *μ*M, and 11.858 *μ*M, respectively (Figure 5(b)). Notably, the number of SR cells in the 12 *μ*g/mL PFGS combined with the Sor group was significantly reduced compared to SR cells at the same concentration (0, 2.5, 5, 10, 20, and 40 *μ*m) of Sor treatment alone, and the CDI values of 12 *μ*g/mL PFGS combined with different concentrations of Sor were 1.02, 0.99, 0.91, 0.70, and 0.65, respectively. The drug combination showed a synergistic effect. The 6 *μ*g/mL PFGS combined with the Sor group had a similar but more limited effect and significantly decreased the number of SR cells (Figure 5(c)). The effect of PFGS combined with Sor on cell invasion ability was evaluated in SR cells by the Transwell assay, in chambers covered with Matrigel. It was shown that after incubation for 24 hours, the invasion of SR cells following treatment with 12 *μ*g/mL PFGS or 5 *μ*m Sor or both drugs combined, in the microporous membrane of the Transwell chamber, was significantly lower compared to the control group (Figure 5(d)). Furthermore, the protein expression of NF-*κ*B p65, HIF-2*α*, and SerpinB3 in SR cells in the Sor, PFGS, and PFGS combined with Sor groups was markedly lower than in the control group (Figure 5(e)). In addition, the expression of these parameters in the PFGS combined with the Sor group was lower than that in the Sor and PFGS groups.

## 4. Discussion

The most frequent clinical malignancy is liver cancer, which is one of the main causes of mortality among cancer patients globally. Sor is the first-line treatment for liver cancer. Sor has been shown in clinical research to improve survival in individuals with liver cancer in various areas, phases, and races [2, 3]. However, resistance due to long-term Sor usage continues to be a serious obstacle in the treatment of HCC patients [9, 21]. Sor-acquired drug resistance is linked to aberrant signaling pathway activation, altered tumor microenvironment, and EMT transformation [9]. Due to the intricacy of resistance mechanisms, a unique strategy is required to increase Sor efficacy in HCC [22]. NF-*κ*B signaling pathway activation lowers the efficacy of numerous anticancer treatments such as chemotherapy and radiation, and it has been demonstrated to play a crucial role in Sor desensitization in liver cancer [23]. The impact of Sor on NF-*κ*B was discovered to be conflicting. Expression of the NF-*κ*B p65 protein was greater in Sor desensitized hepatoma cells than in normal hepatoma cells in our study, which was consistent with earlier research. On the one hand, Sor may decrease NF-*κ*B expression and restrict nuclear translocation [24, 25], but it can also trigger I*κ*B-independent activation of the NF-*κ*B pathway [26]. Recent research has shown that inhibition of NF-*κ*B pathway activation can significantly improve Sor efficacy in HCC, Hep3B, or SR cells [11, 27]. As a result, combining NF-*κ*B pathway inhibitors is a feasible technique for sensitizing HCC to Sor.

Shaoyao Ruangan Mixture is a traditional Chinese medicine preparation, it is prepared in our hospital and is based on a traditional Chinese medicine prescription that has been used clinically for over 20 years with no adverse effects and great safety [14]. A previous study demonstrated that Shaoyao Ruangan Mixture had the capability to produce antitumor effects by inhibiting NF-*κ*B expression [16], the main active components detected by HPLC included paeoniflorin (PF) and geniposide (GS) [28], which are the main active ingredients of White Peony Root and Fructus Gardeniae Praeparatus [29]. Modern research has revealed that PF or GS has anti-inflammatory and antitumor action, as well as the ability to reduce multidrug resistance of chemotherapeutic medications by dramatically inhibiting NF-*κ*B and its upstream and downstream targets [17–19]. As a result, we believe that PFGS is the main medicinal component in Shaoyao Ruangan Mixture that inhibits NF-*κ*B and may enhance the antiliver tumor effect of Sor. Moreover, PFGS is a clear monomer mixture that not only embodies the compatibility theory of the assistance of traditional Chinese medicine but also simplifies the research goal of the complex components of the Shaoyao Ruangan Mixture. Therefore, we examined the antihepatoma effects of PFGS combined with Sor. We discovered that PFGS could not only synergistically increase the efficacy of Sor in H22 tumor-bearing mice, enhance the hepatoma cell proliferation inhibition and apoptosis-promoting action of Sor in HCC tissue, but can also restore SR cell sensitivity to Sor and minimize SR cell invasion. These findings demonstrated that combining PFGS and Sor had considerably enhanced effects in both normal and drug-resistant HCC, suggesting that the anti-HCC action of Sor might be greatly boosted by PFGS. Therefore, we continued to investigate the mechanism of PFGS in enhancing Sor's antitumor activity.

It has been observed in recent years that NF-*κ*B could diminish HCC sensitivity to Sor by increasing activation of the HIF-2*α*/SerpinB3 signaling pathway [30, 31]. The stress response generated by hypoxia in tumor tissue during Sor treatment can boost the development and activation of HIF-2*α*, which promotes aberrant tumor cell metabolism and leads to desensitization to Sor [32, 33]. HIF-2*α* expression and nuclear accumulation are controlled by NF-*κ*B. HIF-2*α* has a particular binding site for NF-*κ*B in its promoter region and activated NF-*κ*B can increase protein stabilization and nuclear translocation [34, 35]. It was discovered that activating NF-*κ*B in HCC cells resulted in a long-term elevation in HIF-2*α* levels, which consequently lowered the susceptibility of hepatoma cells to Sor [36, 37]. In addition, activated HIF-2*α* can connect to the SerpinB3 promoter and enhance molecule synthesis, transcription, and expression [38, 39].

SerpinB3 is a serine protease inhibitor, which is not detectable in normal hepatocytes and has been found progressively upregulated in liver cirrhosis, dysplastic nodules, and hepatocellular carcinoma [40]. The effects of SerpinB3 have been reported to include induced neoplastic cell apoptosis resistance, increased neoplastic cell proliferation, and triggered EMT which has been proposed to also contribute to increased invasiveness of cancer cells and to the development of metastasis and cancer progression [41]. SerpinB3 expression was highly elevated in primary liver cancer tissues with a high degree of malignancy, and SerpinB3 plays an important role in the upregulation of TGF-*β*, which mediated EMT and contributes to sorafenib resistance in HCC cells [42]. Recently, a study clearly showed that hepatocellular carcinoma cells overexpressing SerpinB3 are more resistant to Sor treatment, and was associated with the activation of caspase signaling [31].

Thus, the degree of activation of the NF-*κ*B/HIF-2*α*/SerpinB3 pathway influenced HCC susceptibility to Sor. In this investigation, we discovered that PFGS combined with Sor could significantly decrease activation of the NF-*κ*B/HIF-2*α*/SerpinB3 pathway in H22 tumor-bearing mice and SR cells compared with a single treatment, which led to increased antitumor activity, reduced invasion ability of hepatoma cells, and perhaps prevented EMT in hepatoma cells, although further study is needed to validate this. The above results on the NF-*κ*B/HIF-2*α*/SerpinB3 pathway provide the rationale for PFGS combined with Sor for the treatment of hepatocellular carcinoma.

## 5. Conclusion

Sor has demonstrated considerable therapeutic benefits in animals and clinical trials, and the US Food and Drug Administration has authorized its use in clinical liver cancer first-line treatment [43]. However, drug resistance to Sor can enhance the proliferation and invasion of liver cancer cells. Effective control of Sor resistance has become critical for achieving better therapeutic benefits. We found that PFGS effectively enhanced the inhibitory effect of Sor on tumor proliferation and invasion and promoted tumor apoptosis, resulting in a significantly increased antitumor effect of Sor *in vivo* and *in vitro* by suppression of the NF-*κ*B/HIF-2*α*/SerpinB3 pathway. Our findings supported the use of Sor in combination with PFGS or Shaoyao Ruangan Mixture to treat HCC patients. However, our research had many limitations. Due to insufficient funds and other reasons, we did not study the antitumor effect and mechanism of PFGS combined with Sor in Sor-resistant cell tumor-bearing mice. Thus, the *in vitro* research results lacked *in vivo* experimental verification. In addition, to minimize the impact of individual differences on the experimental results, we used only male mice to establish the tumor-bearing model. Therefore, our experimental results may be biased in female animal models. The above limitations in this study will be the goal of our continued research in the future. Our research findings provide a new theoretical and experimental basis for the clinical application of PFGS combined with Sor in the treatment of liver cancer, enrich the research foundation of traditional Chinese medicine combined with Sor in the treatment of liver cancer, and benefit liver cancer patients.

## Data Availability

The data used to support the findings of this study are available from the corresponding author upon request.

## References

[1] Sung H., Ferlay J., Siegel R. L. (2021). Global cancer statistics 2020: GLOBOCAN estimates of incidence and mortality worldwide for 36 cancers in 185 countries. *CA: A Cancer Journal for Clinicians*.

[2] Bruix J., Cheng A.-L., Meinhardt G., Nakajima K., De Sanctis Y., Llovet J. (2017). Prognostic factors and predictors of sorafenib benefit in patients with hepatocellular carcinoma: analysis of two phase III studies. *Journal of Hepatology*.

[3] Kudo M., Arizumi T. (2017). Transarterial chemoembolization in combination with a molecular targeted agent: lessons learned from negative trials (Post-TACE, BRISK-TA, SPACE, ORIENTAL, and TACE-2). *Oncology*.

[4] Sun T., Liu H.-c., Liang M. (2017). Multiple roles of autophagy in the sorafenib resistance of hepatocellular carcinoma cell. *Physiol Biochem*.

[5] Zhu Y.-j., Zheng B., Wang H.-y., Chen L. (2017). New knowledge of the mechanisms of sorafenib resistance in liver cancer. *Acta Pharmacologica Sinica*.

[6] Zhang W., Sun H.-c., Wang W.-q. (2012). Sorafenib down-regulates expression of HTATIP2 to promote invasiveness and metastasis of orthotopic hepatocellular carcinoma tumors in mice. *Gastroenterology*.

[7] Wang H.-y., Xu L., Zhu X.-y., Wang P., Chi H., Meng Z. (2014). Activation of phosphatidylinositol 3-kinase/Akt signaling mediates sorafenib-induced invasion and metastasis in hepatocellular carcinoma. *Oncology Reports*.

[8] Procopio G., Apollonio G., Cognetti F. (2019). Sorafenib versus observation following radical metastasectomy for clear-cell renal cell carcinoma: results from the phase 2 randomized open-label RESORT study. *European Urology Oncology*.

[9] Taniguchi K., Karin M. (2018). NF-*κ*B, inflammation, immunity and cancer: coming of age. *Nature Reviews Immunology*.

[10] Hu B., Xu Y., Li Y.-C. (2020). CD13 promotes hepatocellular carcinogenesis and sorafenib resistance by activating HDAC5-LSD1-NF-*κ*B oncogenic signaling. *Clinical and Translational Medicine*.

[11] Yu J.-Q., Wang N.-Z., Gong Z.-Q (2021). Cytochrome P450 1A2 overcomes nuclear factor kappa B-mediated sorafenib resistance in hepatocellular carcinoma. *Oncogene*.

[12] Lo J., Lau E. Y. T., Ching R. H. H. (2015). Nuclear factor kappa B-mediated CD47 up-regulation promotes sorafenib resistance and its blockade synergizes the effect of sorafenib in hepatocellular carcinoma in mice. *Hepatology*.

[13] Guan Z., Ding C., Du Y. (2014). HAF drives the switch of HIF-1*α* to HIF-2*α* by activating the NF-*κ*B pathway, leading to malignant behavior of T24 bladder cancer cells. *International Journal of Oncology*.

[14] Zhen H., Qian X., Fu X., Chen Z., Zhang A., Shi L. (2019). Regulation of Shaoyao ruangan mixture on intestinal flora in mice with primary liver cancer. *Integrative Cancer Therapies*.

[15] Zhang H.-Y., Jiang J.-W., Ni M. W., Peng Y., He F. (2014). The inhibitory effect of Shaoyao ruangan formula on mice with transplanted H22 hepatocarcinoma and its mechanism research. *Journal of Cancer Research and Therapeutics*.

[16] Li J.-f., Zheng X.-r., Jiang J.-w. (2021). Study of the inhibitory effect of Shaoyao Ruangan Formula on the growth of liver cancer in nude mice based on Bax/Bcl-2-Caspase-3 pathway and its mechanism. *China Modern Doctor*.

[17] Wu X.-X., Huang X.-L., Chen R.-R. (2019). Paeoniflorin prevents intestinal barrier disruption and inhibits lipopolysaccharide (LPS)-Induced inflammation in caco-2 cell monolayers. *Inflammation*.

[18] Song S.-l., Xiao X.-y., Guo D. (2017). Protective effects of paeoniflorin against AOPP-induced oxidative injury in HUVECs by blocking the ROS-HIF-1*α*/VEGF pathway. *Phytomedicine*.

[19] Jiang H.-W, Ma Y.-J., Yan J.-Q., Liu J., Li L. (2017). Geniposide promotes autophagy to inhibit insulin resistance in HepG2 cells via P62/NF *κ*b/GLUT4. *Molecular Medicine Reports*.

[20] Luo JY., Liao JB., Wang YM. (2022). Advances in traditional Chinese medicine for liver disease therapy in 2021. *Traditional Medical Research*.

[21] Guo Z.-g., Cao M.-q., You A. (2016). Metformin inhibits the prometastatic effect of sorafenib in hepatocellular carcinoma by upregulating the expression of TIP30. *Cancer Science*.

[22] Galmiche A., Chauffert B., Barbare J. C. (2014). New biological perspectives for the improvement of the efficacy of sorafenib in hepatocellular carcinoma. *Cancer Letters*.

[23] Li F., Sethi G. (2010). Targeting transcription factor NF-*κ*B to overcome chemoresistance and radioresistance in cancer therapy. *Biochimica et Biophysica Acta (BBA)-Reviews on Cancer*.

[24] Chen J. C. H., Chuang H. Y., Hsu F. T., Chen Y. C., Chien Y. C., Hwang J. J. (2016). Sorafenib pretreatment enhances radiotherapy through targeting MEK/ERK/NF-*κ*B pathway in human hepatocellular carcinoma-bearing mouse model. *Oncotarget*.

[25] Li J., Zhou Y., Liu Y. (2018). Sorafenib inhibits caspase-1 expression through suppressing TLR4/stat3/SUMO1 pathway in hepatocellular carcinoma. *Cancer Biology & Therapy*.

[26] Dudgeon C., Peng R., Wang P., Sebastiani A., Yu J., Zhang L. (2012). Inhibiting oncogenic signaling by sorafenib activates PUMA via GSK3*β* and NF-*κ*B to suppress tumor cell growth. *Oncogene*.

[27] Wu J.-M., Sheng H.-m., Saxena R. (2009). NF-*κ*B inhibition in human hepatocellular carcinoma and its potential as adjunct to sorafenib based therapy. *Cancer Letters*.

[28] Lu B.-Z., Li X.-H., Yang J.-F. (2001). Determination of paeoniflorin and geniposide in Zhonggan oral liquid by HPLC. *Chinese Pharmaceutical Journal*.

[29] Liao K., Gong L. Y., Yang Y. (2022). A comprehensive review of research progress in Chinese medicines for primary liver cancer treatment. *Traditional Medical Research*.

[30] Ishibashi K., Koguchi T., Matsuoka K. (2018). Interleukin-6 induces drug resistance in renal cell carcinoma. *Fukushima Journal of Medical Science*.

[31] Turato C., Fornari F., Pollutri D. (2019). MiR-122 targets SerpinB3 and is involved in sorafenib resistance in hepatocellular carcinoma. *Journal of Clinical Medicine*.

[32] He C.-J., Sun X. P., Qiao H.-Q. (2012). Downregulating hypoxia-induciblefactor-2*α* improves the efficacy of doxorubicin in the treatment of hepatocellular carcinoma. *Cancer Science*.

[33] Zhao J., Du F., Shen G., Zheng F., Xu B. (2015). The role of hypoxia-induciblefactor-2 in digestive system cancers. *Cell Death & Disease*.

[34] Wang X.-c., Dong J., Jia L. (2017). HIF-2-dependent expression of stem cell factor promotes metastasis in hepatocellular carcinoma. *Cancer Letters*.

[35] Méndez-Blanco C., Fondevila F., García-Palomo A., Gonzalez-Gallego J., Mauriz J. L. (2018). Sorafenib resistance in hepatocarcinoma: role of hypoxia-inducible factors. *Experimental and Molecular Medicine*.

[36] Ma L., Li G.-x., Zhu H.-q. (2014). 2-Methoxyestradiol synergizes with sorafenib to suppress hepatocellular carcinoma by simultaneously dysregulating hypoxia-induciblefactor-1 and -2. *Cancer Letters*.

[37] Green Y. S., Sargis T., Reichert E. C. (2019). Hypoxia-associated factor (HAF) mediates neurofibromin ubiquitination and degradation leading to ras-ERK pathway activation in hypoxia. *Molecular Cancer Research*.

[38] Cannito S., Turato C., Paternostro C. (2015). Hypoxia up-regulates SERPINB3 through HIF-2*α* in human liver cancer cells. *Oncotarget*.

[39] Cannito S., Foglia B., Villano G. (2019). SerpinB3 differently up-regulates hypoxia inducible factors -1*α*and-2*α* in hepatocellular carcinoma: mechanisms revealing novel potential therapeutic targets. *Cancers*.

[40] Pontisso P. (2014). Role of SERPINB3 in hepatocellular carcinoma. *Annals of Hepatology*.

[41] Quarta S., Vidalino L., Turato C. (2010). SERPINB3 induces epithelial-mesenchymal transition. *The Journal of Pathology*.

[42] Turato C., Vitale A., Fasolato S. (2014). SERPINB3 is associated with TGF-*β*1 and cytoplasmic *β*-catenin expression in hepatocellular carcinomas with poor prognosis. *British Journal of Cancer*.

[43] Pascual S., Herrera I., Irurzun J. (2016). New advances in hepatocellular carcinoma. *World Journal of Hepatology*.

